# Exposure to hate speech deteriorates neurocognitive mechanisms of the ability to understand others’ pain

**DOI:** 10.1038/s41598-023-31146-1

**Published:** 2023-03-13

**Authors:** Agnieszka Pluta, Joanna Mazurek, Jakub Wojciechowski, Tomasz Wolak, Wiktor Soral, Michał Bilewicz

**Affiliations:** 1 Faculty of Psychology, University of Warsaw, Stawki 5/7 Street, 00-183 Warszawa, Poland; 2 Bioimaging Research Center, World Hearing Center of Institute of Physiology and Pathology of Hearing, Warszawa, Poland; 3 Laboratory of Emotions Neurobiology, Nencki Institute of Experimental Biology PAS, Warsaw, Poland

**Keywords:** Neuroscience, Psychology, Human behaviour

## Abstract

The widespread ubiquity of hate speech affects people's attitudes and behavior. Exposure to hate speech can lead to prejudice, dehumanization, and lack of empathy towards members of outgroups. However, the impact of exposure to hate speech on empathy and propensity to attribute mental states to others has never been directly tested empirically. In this fMRI study, we examine the effects of exposure to hate speech on neural mechanisms of empathy towards ingroup (Poles) versus outgroup members (Arabs). Thirty healthy young adults were randomly assigned to 2 groups: hateful and neutral. During the fMRI study, they were initially exposed to hateful or neutral comments and subsequently to narratives depicting Poles and Arabs in pain. Using whole-brain and region of interest analysis, we showed that exposure to derogatory language about migrants attenuates the brain response to someone else’s pain in the right temporal parietal junction (rTPJ), irrespective of group membership (Poles or Arabs). Given that rTPJ is associated with processes relevant to perspective-taking, its reduced activity might be related to a decreased propensity to take the psychological perspective of others. This finding suggests that hate speech affects human functioning beyond intergroup relations.

## Introduction

The spread of hate speech, a derogatory language based on racial, religious, or sexual prejudice, has become one of the key challenges for contemporary societies^1–3^. According to the epidemic model of hate speech^4^, people exposed to hate speech in their environment become prone to use derogatory language and engage in other forms of intergroup discrimination due to normative, emotional, and behavioural transitions caused by frequent exposure to such language.

As repeated exposure to violence reduces people’s emotional responsiveness and decreases empathy when observing subsequent violent actions^5–8^, it is plausible that a similar process could be observed among people continuously exposed to verbal violence^6,7^.

Indeed, initial survey and experimental results^9^ suggest that hate speech leads to desensitization, and this in turn affects intergroup relations. Conversely, recent findings suggest that empathy-inducing interventions can successfully resensitize individuals to hate speech and make them less supportive of the use of such statements^10^.

Empathy is a multifaceted construct referring to the human’s capacity to share and understand others' feelings that are induced by the perception of another person's affective state e.g. pain^11^. Its elicitation is modulated by many interrelated factors^12^. For example, lack of empathy toward outgroup members has been observed in several empirical studies concerning intergroup empathy bias^13,14^ and parochial empathy^15,16^. Although these phenomena seem to be fundamental aspects of intergroup relations, it is possible that specific social pressures could enhance them, leading to empathic failure^17^. Hate speech could be considered one of the factors enhancing empathic failure in intergroup relations.

Another stream of research on intergroup interaction has focused on the ability to attribute mental states—beliefs, intents, desires, emotions, and knowledge—to ourselves and others, referred to as theory of mind (ToM) or mentalizing^18^. Studies on dehumanized perception found that people watching members of groups that are considered lacking both warmth and competence react with lower mentalization^19–21^. This suggests that a specific form of hate speech that dehumanizes certain groups could particularly deteriorate the ability to understand mental states of others.

Lastly, the overexposure to aggression (in e.g. media) and suffering of others may desensitize people to violence as well as to pain of others, leading to compassion collapse—the condition which is marked by psychic fatigue, reduced empathy and feeling detached with others^8,22–24^. Applying this model to hate speech, one could also expect that exposure to such forms of verbal violence could deteriorate people’s ability to empathize and/or mentalize resulting in a more general empathic numbing—a state of depletion in which compassion cannot be generated towards other people.

These three perspectives offer different predictions about the potential effects of exposure to hate speech on the ability to share and understand the mental states of others. According to the empathic failure model, hate speech should enhance existing biases in empathy by increasing empathy toward ingroup members and reducing outgroup-directed empathy. According to the dehumanized perception model, hate speech should reduce the mentalization propensity toward dehumanized outgroups, but should not affect empathy towards ingroup members. According to the empathic numbing hypothesis, exposure to hate speech should limit the ability to empathize (or/and mentalize) with other people, regardless of their group membership.

The behavioral and self-report character of previous studies did not allow the identification of the specific neurocognitive mechanism that would link empathy and/or mentalizing abilities to the use of hate speech. In this study, we seek to answer this question using a social neuroscience approach and functional magnetic resonance imaging (fMRI) techniques.

Although both empathy and ToM are multidimensional and often intertwined abilities that are jointly required in many social situations, they are rooted in separable brain networks^25^. ToM involves an extended neural network (collectively referred to as the ToM network) comprising the temporoparietal junction (TPJ), precuneus (PC), posterior superior temporal sulcus (pSTS), medial prefrontal cortex (MPFC), and temporal poles^26–28^. Among them, the TPJ (especially in the right hemisphere) is considered to play a critical role in processing information about someone else’s psychological perspective. Studies that have examined the neural basis of empathy have identified regions, such as the anterior cingulate (ACC) cortex^29^ and anterior insula (AI)^30^ (collectively referred to as the pain matrix) that may be essential for processing information about vicarious experiences of other people. An accumulating body of evidence shows that the ACC and the AI respond to both first- and third-person experiences of pain. Therefore, this response pattern might indicate “state-matching” between the observer and target^31^.

Importantly, a number of studies have shown that people process information about the affective and cognitive mental states of members of distinct ethnic or cultural groups differently^32^. For example, it has been demonstrated that people show diminished brain activity of the ACC in response to physical pain experienced by others of different ethnicities^33^. In contrast, the study of Marthur and colleagues^34^ demonstrated that while confronted with the suffering of the victims of hurricane Katrina, African–Americans and Caucasian–Americans differently activated the mentalizing network (specifically MPFC) but not the pain matrix. The brain response varied depending on the victim's ethnicity.

These neuropsychological differences in reactions to ingroup and outgroup members' emotional or physical suffering may be further amplified in contexts dominated by hate speech. Surprisingly, no studies we know have directly examined the impact of exposure to hateful statements (hate speech) on the neurocognitive processes.

In the present study, we aimed to disentangle three potential consequences of exposure to hate speech (e.g. the empathic failure, the dehumanized perception, the empathic numbing) by examining neurocognitive mechanisms of empathy and ToM. To this end, we conducted a study of Polish individuals and chose Arabs as the outgroup because in recent times, this group has been subject to political attacks in the Polish media^35^. While in the magnetic resonance 3 T scanner, individuals were presented with long lists of negative online comments about the outgroup (hateful condition) or not (neutral condition), interspersed with painful and non-painful stories featuring protagonists of Polish (ingroup) and Arab (outgroup) ethnicities.

Therefore, we aimed to test the following hypotheses:

H1. According to the empathic failure model, exposure to hate speech leads to decreased brain activity within the AI and ACC in response to outgroup members’ pain or suffering, and increased activity within these areas in response to ingroup members’ pain or suffering.

H2. According to the dehumanized perceptions model, exposure to hate speech leads to attenuation of the ToM network in response to outgroup (but not ingroup) members’ pain or suffering.

H3. According to the empathic numbing hypothesis, exposure to hate speech leads to decreased activity in the pain matrix and/or ToM network in response to others’ pain or suffering, regardless of their group membership.

## Method and materials

### Participants

Thirty right-handed Polish participants (*M*_*age*_ = 23.7, *SD* = 4.28, females = 18; *M*_*education years*_ = 14.9, *SD* = 2.10) with no reported history of neurological or psychiatric diagnoses, having no contraindications to taking part in magnetic resonance imaging examinations, volunteered to participate in the study from April 2019 to August 2019. Twenty seven participants had declared their ethnic identity as only Polish, 2 described their ethnicity as Polish–American, and 1 as Polish–Japanese. All subjects were born and raised in Poland. They had normal or corrected-to-normal vision, provided written informed consent, and were compensated with a small gift. The experimental procedure was approved by the Ethical Committee of the Faculty of Psychology, University of Warsaw, and was carried out in accordance with the Declaration of Helsinki.

### Procedure

Participants were randomly assigned to two equinumerous groups (*n* = 15 each), each with equal gender proportion (9 women and 6 men), and exposed either to negative comments about the Arabs (hateful) or about current social issues (neutral). The study had a mixed 2 (Type of Comments: hateful vs. neutral) × 2 (Type of Stories: pain vs. no pain) × 2 (Protagonist’s Ethnicity: Polish vs. Arab) design. The Type of Comments was a between-subject factor, while the other two (Stories and Protagonist’s Ethnicity) were within-subject factors. Sensitivity analysis was conducted with G*Power to find a minimum detectable effect (MDE) for a 2 (between) × 4 (within) interaction (with power = 0.80 and ɑ = 0.05). These yielded a MDE value equivalent to Cohen’s *f* = 0.22 or η^2^ = 0.045 (see the power analysis protocol uploaded on the OSF repository: https://osf.io/t8fra).

### Study design

**Figure 1:**
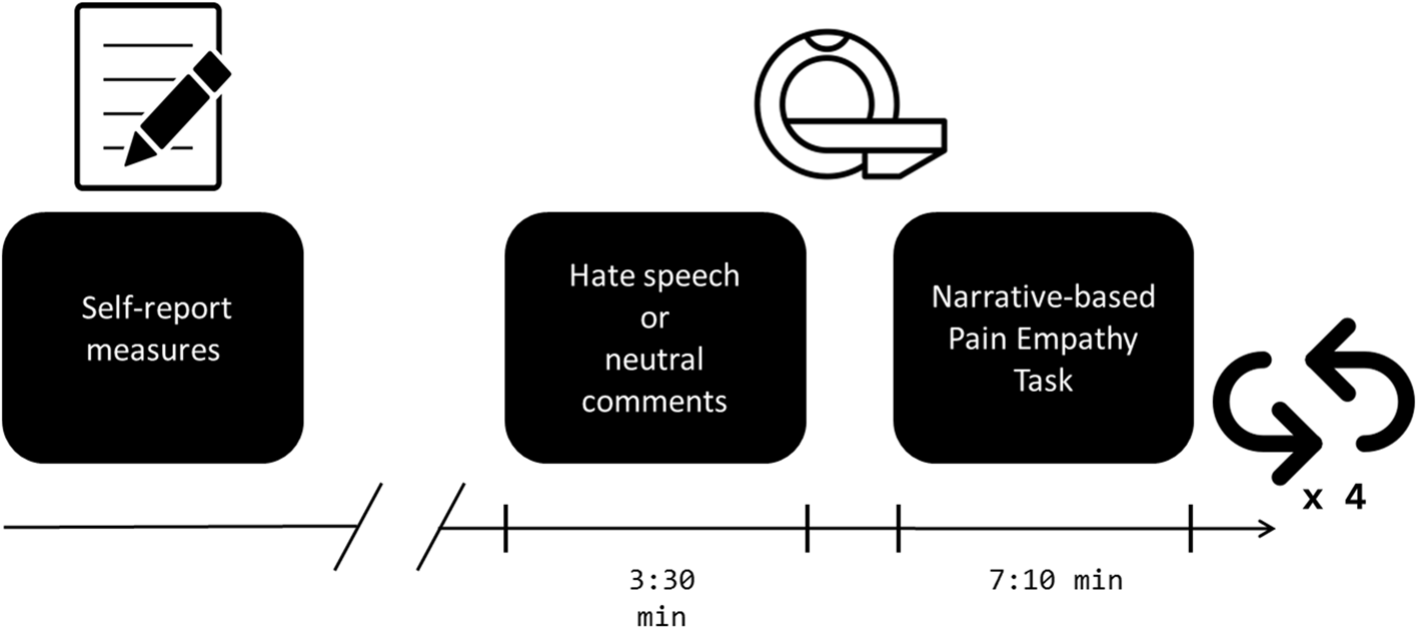
The design of the study.

The whole study consisted of two parts: a self-report measure and fMRI examination (described in the sections below). During the fMRI part, we used an experimental manipulation in the form of internet-like comments (hate speech or neutral), followed by the Narrative-based Pain Empathy Task (described in the sections below). To maintain the impact of the comments during the cognitive task, we used short, consecutive manipulations followed by the task runs repeated four times. The study design is presented in Fig. 1.

### Self-report measures

Two to seven days before fMRI testing, participants completed a questionnaire (administered through Qualtrics Survey Software; Qualtrics, Provo, UT) including demographic variables and two measures of political ideology (right-wing authoritarianism and social dominance orientation) that are known correlates of hate speech attitudes^36^. Right-wing authoritarianism was measured with 6 items (such as “Obedience and respect for authority are the most important values children should learn”) of a shortened version of Funke’s (2005) the three-dimensional Right-Wing Authoritarianism (RWA) scale (⍵_t_ = 0.86, *M* = 3.42, *SD* = 1.25). The Polish adaptation of the Social Dominance Orientation (SDO) scale^37^, shortened to eight items (such as “Superior groups should dominate inferior groups”), was used to assess participants’ preference for social hierarchy over egalitarianism (⍵_t_ = 0.92, *M* = 2.86 *SD* = 1.18). The responses to RWA and SDO were coded on a 7-point scales from 1 (“Definitely disagree”) to 7 (“Definitely agree”).

### Exposure to hateful or neutral comments

In the first part of the procedure, participants in the scanner were exposed to comments (hateful or neutral). The comments were selected based on previous studies on exposure to online hate speech^9^ and were presented in a form imitating real-life internet forums. The comments’ selection procedure was described in detail in the work by Bilewicz and colleagues^38^.

In the hateful comments condition, the comments included derogatory statements directed against Arab and Muslim immigrants and refugees (e.g., “It is high time. This wall should have been put up a long time ago. We need to defend Europe against the hordes”). In the neutral comments condition, the comments focused on current economic and legal issues, as well as international and domestic politics (e.g., “Is it just me who gets pissed off by this neologism: prosumer? Who came up with something so dumb, and why is everyone using it?”). In both conditions, the affective tone of the comments varied from mildly negative to offensive, but the target was different. Note that the label “neutral” means that the comments were not directed against the Arab outgroup (nor any minority group), and not that they were emotionally neutral. The sample comments page can be seen in Supplementary Information Fig. S1. The full list of comments is available on the Open Science Framework (Supplemental Materials A https://osf.io/nw8xq).

Each block in this part consisted of 5 pages of comments (on average three per page) displayed in random order, each for 30 s. A fixation cross (+) was displayed for 20 s in between the comments (C) in the following order: CC + CC + C+. Participants were instructed to read the comments that appeared on the screen and to try to remember them as well as possible. Overall each commentary run lasted 3:30 min. Because we had only 30 unique comments divided into 10 pages, each comment was displayed twice (before I and III as well as II and IV runs of the Narrative-based Pain Empathy Task).

### Narrative-based Pain Empathy Task

After each run of comments, subjects performed a Narrative-based Pain Empathy Task. There were 64 narrative stories used in the study–32 pain stories (describing painful physical sensations e.g. protagonist cut his finger with a sharp knife) and 32 no painful versions of each story. The stories were based on Bruneau and colleagues' experiment (2012) and translated into Polish. The original version and the back-translated versions were compared by a native speaker of Polish, considering the comparability of language and similarity of interpretability. Each of the stories was repeated twice, once with a Polish name and the corresponding picture (above the text) of a protagonist, and the other with an Arab name and the picture of the protagonist^39^. The full list of stories is available on the OSF (Supplemental Materials B https://osf.io/fcnuy/).

The pictures used in the experimental task were faces from the Radboud Faces Database^40^. The database contains 18 Moroccan adult male faces, which were chosen to represent Arab protagonists, and 20 Caucasian adult male faces, which were chosen to represent Polish protagonists. A sample picture can be seen in Supplementary Information Fig. S2.

**Figure 2:**
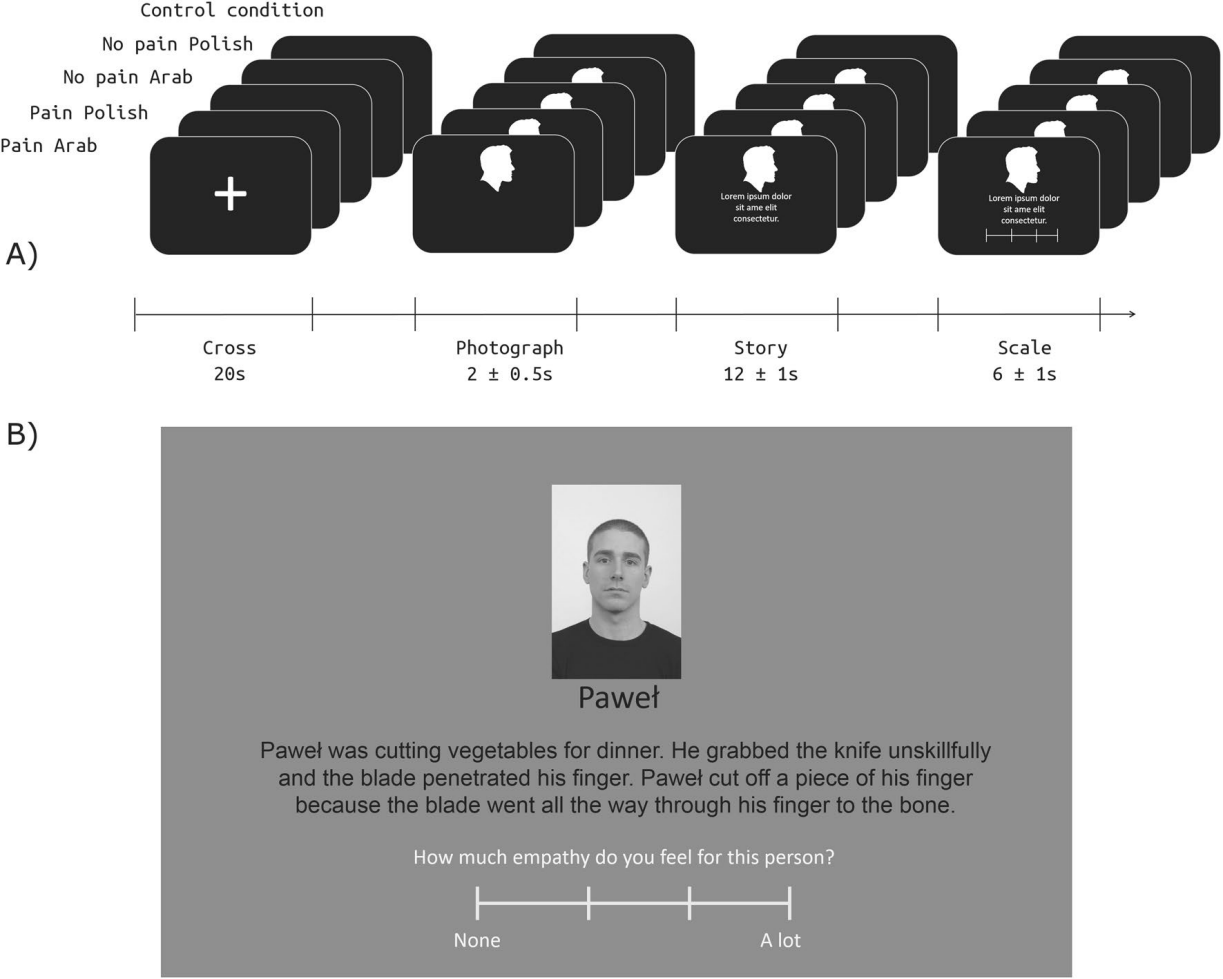
(**A**) A schematic display of the design of the Narrative-based Pain Empathy Task (N-PET) in fMRI. Each experiment condition had the same block structure. In the control condition, the fixation cross was displayed. (**B**) A sample stimulus displayed in the N-PET at the final phase (Scale), when participants were instructed to indicate their empathy towards the protagonist.

Participants were instructed to attentively read the stories accompanied by the names and faces (Photograph). Each story was displayed for 20 s on average (Story). Before each story, a fixation cross was presented for 20 s (Cross). Then, a face picture with the protagonist’s name underneath was displayed for 2 (± 0.5 s jitter) seconds on its own, then the story appeared underneath for an additional 12 s (± 1 s jitter). At the end, a response prompt with a scale appeared underneath the story for 6 s (± 1 s jitter) (Scale). A schematic display of fMRI design with example items of stimuli and a sample page that participants saw in the scanner can be seen in Fig. 2.

After each stimulus, there was an interstimulus interval (ISI) on average for 2 s (± 0.5 s jitter). The prompt asked participants to indicate their empathy towards the protagonist on the following scale: 1 (*none*) to 4 (*a lot*). Participants responded using an MR-safe button box. Average responses and reaction times (RTs) for each condition were computed for each individual. Each run consisted of 16 stories (4 per condition) and 4 fixation crosses, displayed in a pseudorandom order so that no two stimuli of the same type (pain Polish, pain Arab, no pain Polish, no pain Arab, fixation cross) were shown in a row. For each run, a sequence was chosen randomly, and for each stimulus type, the stimuli were assigned randomly without repetition. The whole Narrative-based Pain Empathy Task consisted of 4 runs each lasting 7:10 min. Therefore, the whole fMRI experiment lasted 42:40 min. Stimulus presentation was controlled through a script written in Presentation 21.0 software (Neurobehavioral Systems Inc.) and displayed on MR-compatible full HD display screen (NordicNeurolab Inc.) via a mirror mounted on the head coil.

At the end of the study, the participants were fully debriefed regarding the nature of the study and compensated for their participation with small gifts. Finally, the participants were asked open-ended questions about the Hateful or Neutral Comments to make sure that they had been paying attention to them. Based on these answers, we decided that all participants paid attention to the stimuli and could be included in the further analysis.

### MRI data acquisition

The study took place in the Bioimaging Research Center, Institute of Physiology and Pathology of Hearing, Kajetany, Poland. MRI scanning was performed on a 3 T Siemens Prisma MRI scanner equipped with a 20-channel phased-array RF head coil. Functional data for all tasks were acquired using a Multi-band (Simultaneous Multi-Slice) echo-planar-imaging (EPI) sequence (TR = 1500 ms, TE = 27 ms, flip angle = 90°, FOV = 192 × 192 mm, 94 × 94 mm image matrix, 48 transversal slices of 2.4 mm slice thickness, voxel size of 2.0 × 2.0 × 2.4 mm, Slice Accel. Factor = 2, In-Plane Accel. Factor = 2, IPAT = 4, TA = 7:10 min).

Structural images were collected with a T1-weighted 3D MP-Rage sequence (TR = 2300 ms, TE = 2.26 ms, TI = 900 ms, 8° flip angle, FOV = 208 × 230 mm, image matrix 232 × 256 mm, voxel size of 0.90 × 0.90 × 0.90 mm, 208 slices of 0.90 mm slice thickness, TA = 4:53 min).

### Image processing

Preprocessing and first-level analysis of the fMRI data was carried out with the use of SPM12 (SPM; WellcomeTrust Centre for Neuroimaging, London, UK). The functional data were slice-time corrected, motion corrected, realigned to the first image from each run and co-registered to the individual structural images. High-resolution structural images were segmented and normalized to the common MNI space with resampling to 1 mm isometric voxels. The obtained transformation parameters were applied to the functional volumes with resampling to 2 mm isometric voxels. Spatial smoothing with a Gaussian kernel of full-width half-maximum (FWHM) of 6 mm was performed on the normalized functional images. Additional high-pass filters of 256 s were used for the functional data.

For the first-level statistical analysis, a general linear model was created including a regressor for each condition (pain Polish, pain Arab, no pain Polish, no pain Arab, fixation cross). This resulted in 5 regressors in total. Additionally, six movement parameters (3 translations, 3 rotations) were included as nuisance regressors. The conditions were modeled using a standard boxcar model. For the second-level analysis, we used the Multivariate and Repeated Measures (MRM) toolbox^41^.

To test for a simple effect of pain, at the second level, a two-sample *t*-test was performed using the (pain Arab + pain Polish) > (no pain Arab + no pain Polish) contrast for all participants (*N* = 30). For interaction analysis, images of beta estimates of the experimental conditions from each participant were entered into a three-way repeated measures ANOVA with one between-subject factor: Type of Comments (neutral vs. hateful) and two within-subject factors: Protagonist’s Ethnicity (Polish vs Arabs) and Story Type (pain vs. no pain). Finally, for follow-up post-hoc analysis, voxels from each cluster that showed significant effect/interaction were averaged and then tested with appropriate post-hoc contrasts.

All reported contrasts were thresholded voxel-wise at *p* < 0.001 and family-wise error (FWE) corrected at the cluster level *p*_FWE_ < 0.05. Bspmview toolbox, based on the Anatomy Toolbox, was used for automated anatomical labeling of the results (https://github.com/spunt/bspmview). For visualization of results in surface-based form, BrainNet Viewer was used^42^.

**Figure 3:**
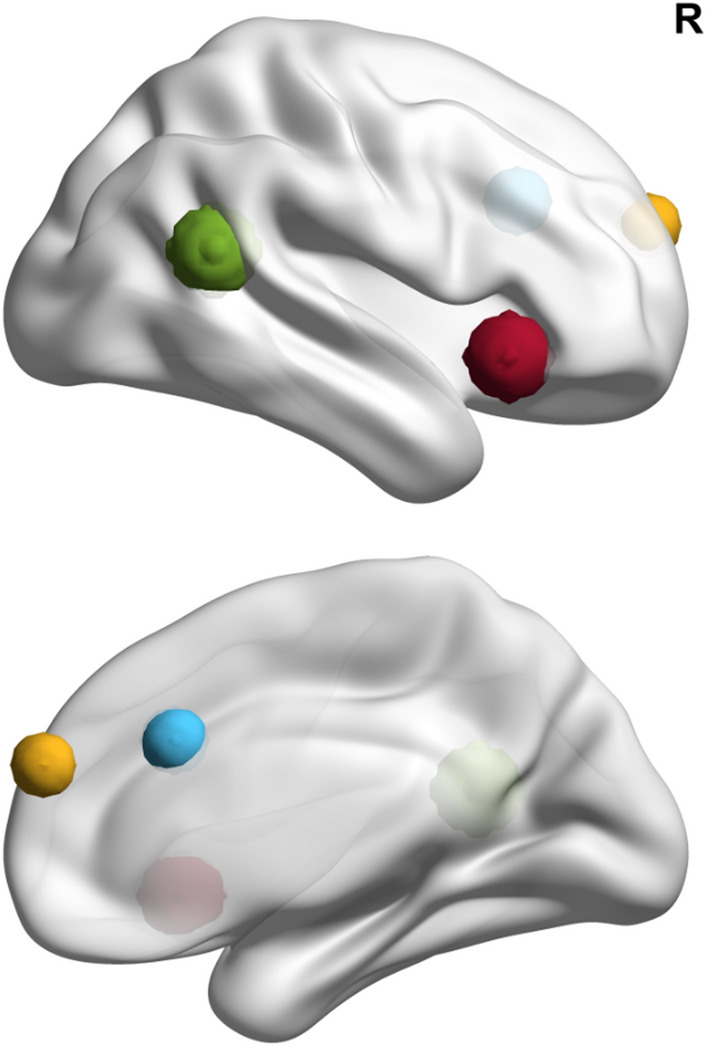
Regions of Interest selected based on Neurosynth survey (only ROIs in the right hemisphere are visualized, ROIs in the left hemisphere were roughly symmetrical, see “fMRI analysis” section for details). AI—red, TPJ—green, dACC—blue, mPFC—yellow.

Subsequently, we conducted Regions of Interest (ROI) analysis based on regions postulated to be involved in empathy, mentalizing, and moral sensitivity in relation to outgroup bias^32^ (AI, ACC, TPJ and mPFC). Neurosynth (Neurosynth.org) with the term “empathy” was queried in order to identify the peak coordinates of the preselected ROIs. This resulted in images from 187 PubMed publications within the regions comprising both the classical “pain matrix” and “ToM matrix” as well brain regions beyond these networks. Finally we created 8 mm spheres centered around the peak coordinates of the left AI [− 34, 22, 0]; right AI [38, 22, − 8], left dorsal ACC (dACC) [− 6, 20, 44], right dACC [10, 24, 30], left TPJ [− 48, − 62, 20], and right TPJ [50, − 50, 18], mPFC [2, 24, 56]. ROIs are presented in Fig. 3.

The averaged beta estimates for each condition of each task from the ROIs were extracted using in-house Matlab scripts.

First, we conducted paired t-tests analysis for pain and no pain conditions for the right and left AI and dACC for each group separately to ensure that painful stories elicited greater BOLD response in the key regions of empathy for physical pain irrespectively of experimental manipulation (neutral vs. hateful).

Finally, a series of ANOVAs was conducted on the following ROIs (selected a priori, see Methods): left and right TPJ, mPFC, left and right insula, and left and right dACC. Each ANOVA followed the same full factorial mixed design, with Type of Comments (neutral vs. hateful, between-subject), the Story Type (no pain vs. pain, within-subject), and Protagonist’s Ethnicity (Arab vs. Polish).

The analyses were carried out using R software(http://www.rstudio.com/).

## Results

### Self-report results obtained during fMRI study

Ratings of empathy towards protagonists in no pain (vs. pain) were low for all participants (the average of median ratings was *M* = 1 and the standard deviation was *SD* = 0). Conversely, average empathy ratings toward protagonists in pain (vs. no pain) were significantly larger than the midpoint of the scale which was 2.5 (*M* = 3.39, *SD* = 0.69), *t*(29) = 7.04, *p* < 0.001, *d* = 1.29. Therefore, to avoid issues due to the lack of homogeneity of variances, further analyses in this section were conducted only on ratings toward protagonists in pain. Median empathy ratings for each participant were transformed using the aligned rank method^43^, and then analyzed with factorial mixed-design ANOVA, with the Type of Comments (neutral vs. hateful) as a between-subject factor, and the Protagonist's Ethnicity (Arab vs. Polish) as a within-subject factor. Participants exposed to hateful comments (M = 3.23, SD = 0.75) empathized to a slightly lesser extent than participants exposed to neutral comments (M = 3.55, SD = 0.63). However, the difference was not statistically significant, *F*(1, 28) = 1.62, *p* = 0.213, η_p_^2^ = 0.055. Participants were also more empathic to Polish (M = 3.42, SD = 0.68) than to Arab protagonists (M = 3.37, SD = 0.74), but also in this case the difference was not statistically significant, *F*(1, 28) = 0.95, *p* = 0.337, η_p_^2^ = 0.033. The interaction of the Type of Comments and the Protagonist’s Ethnicity of the was not significant, *F*(1, 28) = 0.62, *p* = 0.438, η_p_^2^ = 0.022. In a similar vein, analyses of log-transformed RTs in mixed design ANOVA (Type of Comments x Protagonist’s Ethnicity) revealed no significant effect or interactions. Specifically, the main effect of Type of Comments on RT was not significant, *F*(1, 28) = 2.92, *p* = 0.099, η_p_^2^ = 0.090. The main effect of Protagonist’s Ethnicity was not significant, *F*(1, 28) = 1.07, *p* = 0.310, η_p_^2^ = 0.040. The Type of Comments x Protagonist’s Ethnicity interaction was not significant, *F*(1, 28) = 0.002, *p* = 0.963, η_p_^2^ < 0.001.

### Functional MRI results

#### Whole-brain analysis

**Figure 4:**
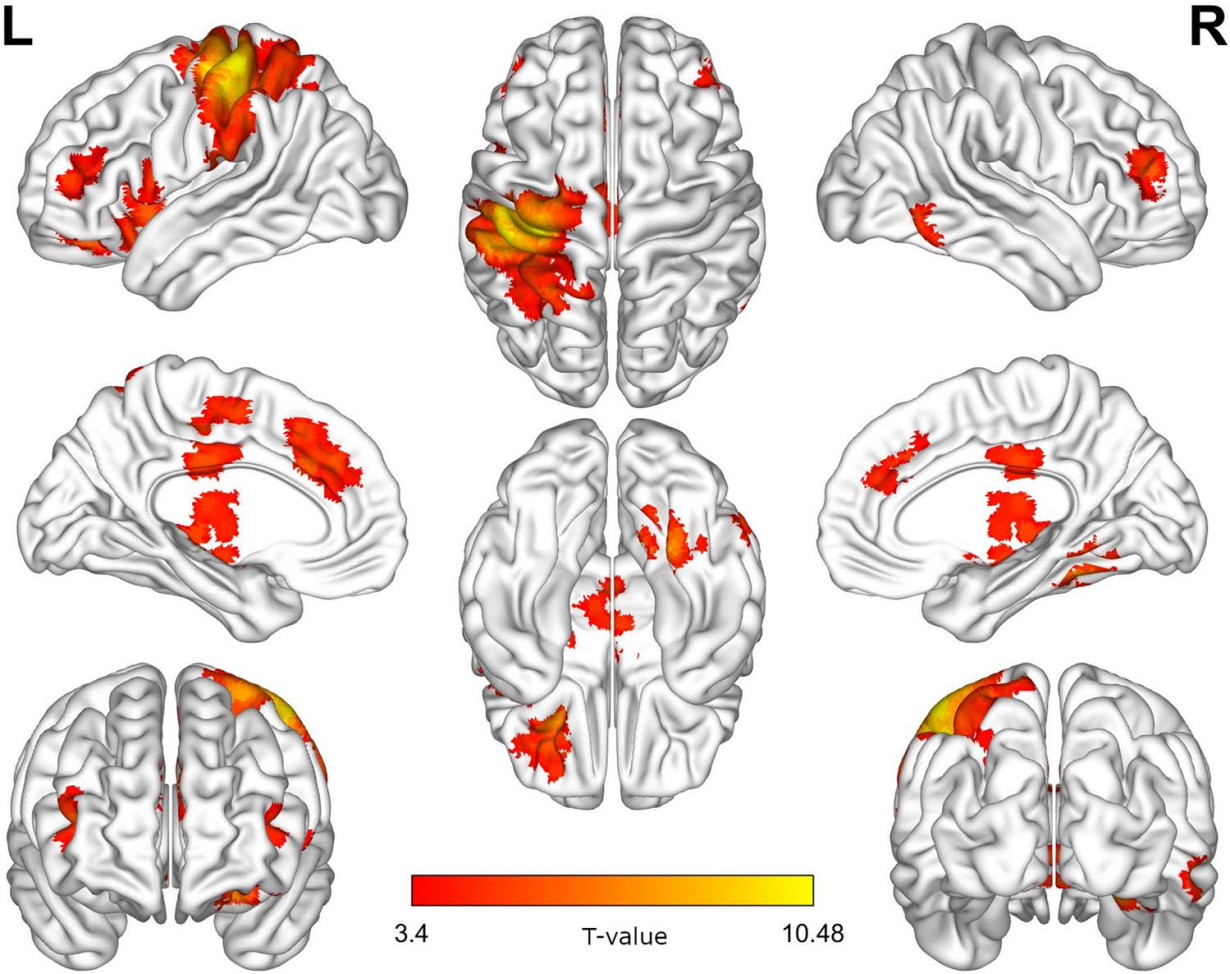
Brain activations (T-map) showing a significant main effect of pain by contrasting conditions: (pain Polish + pain Arab) > (no pain Polish + no pain Arab). Thresholded for voxel-level at *p* < 0.001 and FWE-corrected *p* < 0.05 for cluster size.

The analysis revealed the main effect of Story Type (no pain vs. pain, within-subject). Stories with Pain elicited higher blood oxygen level dependant (BOLD) signal involved in processing information about pain experienced by another person (including the affective components): AI, ACC, pre- and postcentral gyrus, thalamus, caudate, supplementary motor cortex, orbitofrontal cortex, frontal pole bilaterally, cerebellar vermis (4/5). All regions visualized in Fig. 4 are displayed in Table 1.

**Table 1:** Regions showing a significant main effect of pain.

Location	Cluster	T-value	Coordinate (MNI)		
			X (mm)	Y (mm)	Z (mm)
Postcentral gyrus L	4901	10.4812	− 46	− 28	62
Precental gyrus L	4901	7.6525	− 30	− 12	68
Superior parietal gyrus L	4901	5.903	− 30	− 50	64
Lobule IV, V of cerebellar hemisphere R	1242	9.4459	18	− 48	− 22
Lobule IV, V of vermis	1242	5.5222	4	− 62	− 10
Crus II of cerebellar hemisphere R	1242	4.5072	54	− 48	− 40
Caudate nucleus R	872	8.1566	12	14	6
Ventral posterior nucleus of thalamus L	872	6.2541	− 12	− 20	8
Posterior orbital gyrus L	759	7.4245	− 26	28	− 14
Insula L	759	5.88	− 40	10	-4
Middle frontal gyrus L	759	4.6561	− 24	52	− 12
Anterior cingulate cortex, subgenual L	521	6.1257	− 8	24	30
Anterior cingulate cortex, pregenual R	521	4.6454	4	40	24
Middle frontal gyrus R	220	5.7797	40	44	14
Inferior temporal gyrus R	166	5.5933	60	− 64	− 10
Middle cingulate L	266	5.5132	− 2	− 22	32
Supplementary motor area L	266	5.107	− 6	− 6	54
Middle frontal gyrus L	205	5.1466	− 36	36	18
Crus I of cerebellar hemisphere L	189	4.8852	− 34	− 70	− 28

**Figure 5:**
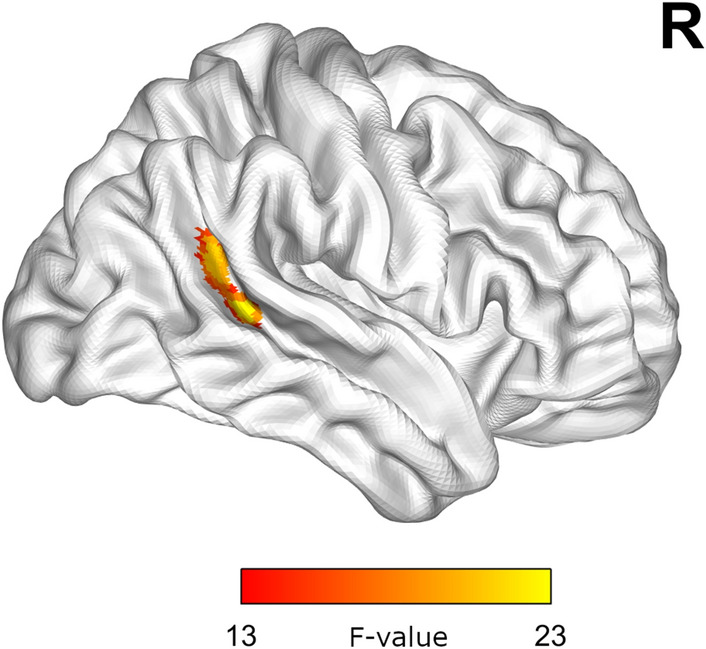
Brain activations (F-map) showing a significant interaction of Type of Comments × Story effect. Thresholded for voxel-level at *p* < 0.001 and FWE-corrected *p* < 0.05 for cluster size.

In addition, there was also a significant Type of Comments × Story interaction in the right TPJ, which is a hub of mentalizing networks (Fig. 5). Interestingly, post-hoc analysis for this region showed that exposure to hateful comments attenuated its activity.

#### Region of interest analysis

Paired t-test analysis yielded that in both groups (hate, neutral) stories with pain were associated with greater BOLD response than stories without pain in the left dACC (hate group: *M*_*pain*_ = 0.51, *SE*_*pain*_ = 0.27 vs. *M*_*nopain*_ = − 0.04, *SE*_*nopain*_ = 0.23, t(14) = 3.66, *p* < 0.05, d = 0.94; neutral group: *M*_*pain*_ = 0.73, *SE*_*pain*_ = 0.28 vs. *M*_*nopain*_ = 0.48, *SE*_*nopain*_ = 0.22, t(14) = 3.28, *p* < 0.05, d = 0.85). In addition, participants exposed to hateful messages reacted with increased BOLD response in right AI (*M*_*pain*_ = 1.01, *SE*_*pain*_ = 0.38 vs. *M*_*nopain*_ = 0.59, *SE*_*nopain*_ = 0.36, t(14) = 2.72, *p* < 0.05, d = 0.7) and participants exposed to neutral comments reacted with higher BOLD response in left AI (*M*_*pain*_ = 0.34, *SE*_*pain*_ = 0.18 vs. *M*_*nopain*_ = 0.16, *SE*_*nopain*_ = 0.16, t(14) = 3.18, *p* < 0.05, d = 0.67) when exposed to pain experienced by the protagonist as compared to no pain stories. This suggests that in both groups the presentation of painful stories resulted in higher BOLD activation in key regions implicated in empathy for physical pain.

**Figure 6:**
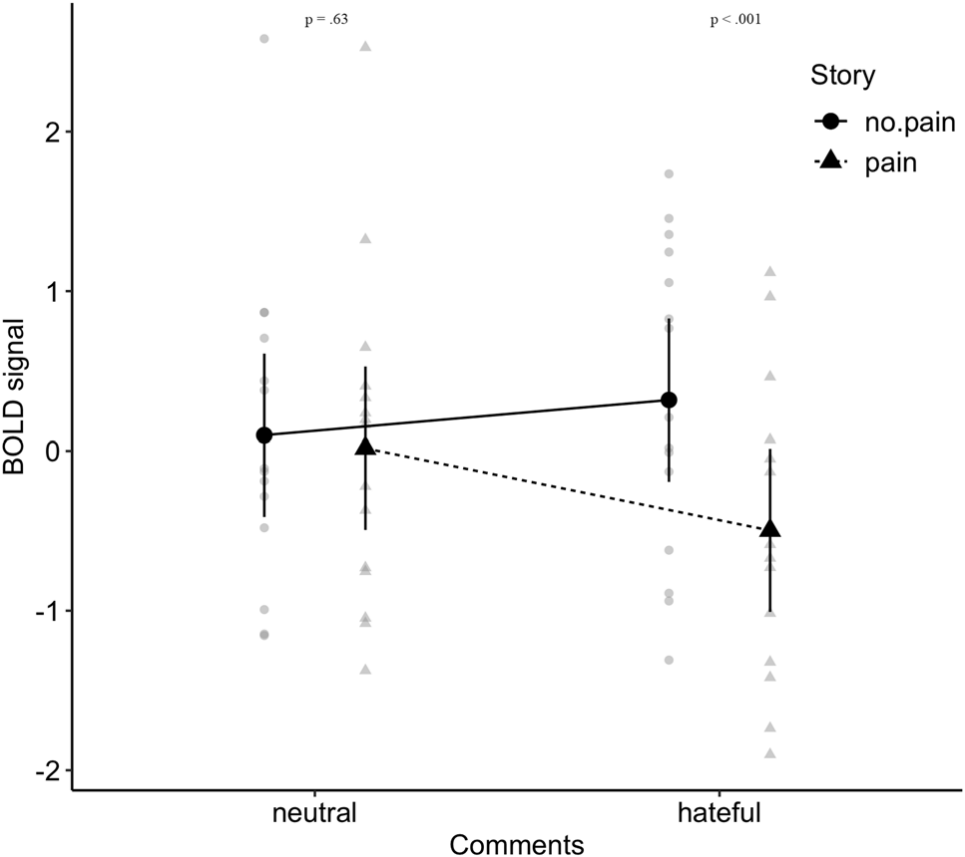
Type of Comments (Neutral vs. Hateful) × Story (No Pain vs. Pain) Interaction Observed in the Right TPJ. Black points indicate estimated means. Gray points indicate individual observations. Vertical lines indicate 95% confidence intervals.

The analyses of ANOVAs conducted on 7 RoIs revealed no significant effects of Type of Comments or Protagonist’s Ethnicity. However, analyses revealed significant main effects of Story. Stories with pain were associated with greater BOLD response than stories without pain in the left insula (*M* = 0.18, *SE* = 0.13 vs. *M* = 0.05, *SE* = 0.14), the right insula (*M* = 0.94, *SE* = 0.23 vs. *M* = 0.70, *SE* = 0.21), the left dACC (*M* = 0.62, *SE* = 0.18 vs. *M* = 0.22, *SE* = 0.16), and the right dACC (*M* = 0.33, *SE* = 0.15 vs. *M* = 0.16, *SE* = 0.15). Conversely, stories with pain were associated with decreased BOLD response in comparison to stories without pain in the left TPJ (*M* = -0.10, *SE* = 0.27 vs. *M* = 0.31, *SE* = 0.28) and right TPJ (*M* = − 0.24, *SE* = 0.18 vs. *M* = 0.21, *SE* = 0.18). However, the effect of the story on the right TPJ was qualified by the significant Type of Comments x Story interaction (see Table 2). As shown in Fig. 6, the difference in signal in the right TPJ between pain and no pain stories observed was significant among participants exposed to hateful comments, *t*(28) = − 4.90, *p* < 0.001, *d* = − 0.81, but not significant among participants exposed to neutral comments, *t*(28) = − 0.49, *p* = 0.63, *d* = − 0.08. Thus, participants exposed to hateful messages reacted with reduced right TPJ activity when exposed to pain experienced by the protagonist as compared to no pain stories. This suggests that among participants exposed to hateful (but not neutral comments), painful stories resulted in lower activation of the right TPJ, irrespective of the protagonist’s ethnicity.

**Table 2:** Results of mixed design analyses of variance for selected regions of interest.

Effect	Left TPJ		Right TPJ		Left insula		Right insula		Left dACC		Right dACC	
	F	η_p_^2^	F	η_p_^2^	F	η_p_^2^	F	η_p_^2^	F	η_p_^2^	F	η_p_^2^
C	0.92	0.028	0.20	0.006	0.11	0.003	0.00	< 0.001	1.30	0.039	0.83	0.025
E	0.18	< 0.001	1.08	0.001	0.00	< 0.001	0.05	< 0.001	0.79	0.001	0.07	< 0.001
C × E	0.03	< 0.001	0.46	< 0.001	0.44	0.001	0.87	< 0.001	1.40	0.002	0.46	< 0.001
S	7.36*	0.018	14.52***	0.050	6.24*	0.008	6.11*	0.010	22.49***	0.045	5.95*	0.010
C × S	0.45	0.001	9.75**	0.034	3.29	0.004	3.48	0.006	3.32	0.007	0.10	< 0.001
E × S	0.03	< 0.001	0.09	< 0.001	3.41	0.007	3.97	0.002	1.62	0.001	3.33	0.003
C × E × S	1.52	0.002	1.65	0.003	0.02	< 0.001	0.00	< 0.001	0.83	< 0.001	0.65	< 0.001

N = 30. Degrees of freedom are 1 and 28 for all effects. C = Comments (neutral vs. hateful), E = Ethnicity (Arab vs. Polish), S = Story (no pain vs. pain).

*p < 0.05 *p < 0.01 ***p < 0.001.

None of the remaining two-way or three-way interactions were significant. For mPFC none of the effects were statistically significant. In all, ROI analysis (on regions identified based on the query in Neurosynth) was consistent with the whole brain analysis.

## Discussion

This experimental study tested the effects of prior hate speech exposure on neurocognitive mechanisms of empathic reaction and/or propensity to attribute mental states to others when contemplating another’s pain in an intergroup setting. Building our predictions on previous research on intergroup bias in empathy, as well as desensitization to verbal aggression, in this study, we investigated 3 possible theoretical models: the empathic failure model, the dehumanized perception model, and the empathic numbing model.

The results demonstrate that exposure to hate speech, even for about a quarter of an hour, affects brain response by reducing BOLD response in the region involved in mentalizing (rTPJ) while faced with the pain of others, regardless of the group membership of the protagonist (Polish or Arab).

Firstly, whole-brain analysis revealed that the narrative scenarios used in the fMRI experiment activate brain regions typically considered as the core empathy network: AI, ACC, SMA, pre- and postcentral gyrus, parietal lobe, thalamus, frontal pole (specifically the inferior frontal gyrus)^44^. The ROI analysis was in line with the whole-brain results and showed increased activity within the insula and dACC bilaterally in response to painful stimuli. These results confirm that stimuli depicting another person's pain lead to the recruitment of the pain matrix regions as planned.

Secondly, neither fMRI nor self-report measures collected during the fMRI session were indicative of in-group biases. On the one hand, the lack of intergroup bias in empathy ratings may be related to the fact that self-report assessment during the fMRI study was done on a 4-point scale, which may not have been sensitive enough to capture possible subtle ingroup bias. These results are also consistent with the study of Bruneau and colleagues^45^, which found that being witness to physical pain experienced by distant outgroups evokes the same level of compassion, as well as similar neural activity at the whole-brain level within the pain matrix, as observing the pain of members of one's own group. Given that Poles have not been involved in ongoing conflicts with Arabs, do not have a history of conflict with them, and that the two groups come from very different geographic, linguistic, and political backgrounds, Arabs conform to the definition of a distant outgroup for Poles.

This result might be also explained by the participants' sociodemographic characteristics (young well-educated adults who have had an average of 14.9 years of schooling) and ideological orientation. Given that a preference for hierarchy has been shown to be associated with reduced empathy towards outgroup persons^46^, participants’ moderate RWA and SDO scores could possibly explain the absence of parochial empathy (i.e., the tendency to show greater empathic concern for members of one’s ingroup) in the studied group.

Finally, both whole-brain and independent ROI analysis revealed that exposure to derogatory language about migrants and minority groups suppresses the brain response of the rTPJ. This effect occurred regardless of the group membership of the protagonist (Polish or Arab). A sizable number of studies have shown the rTPJ to be robustly activated when people attribute mental states to others (intentions/beliefs), in order to explain or predict or make moral judgements about their actions^47,48^. Interestingly, it has also been demonstrated that disrupting one’s rTPJ using TMS impairs the ability to consider one's beliefs and intentions when making moral judgments^49^. Therefore, reduction of rTPJ activity after exposure to hate speech might reduce the propensity to project ourselves into the reality of others in order to share/understand their psychological state. Given that the ability to adopt others’ perspectives is an essential component of compassionate responses leading to altruistic behaviors^50^, the observed effect might have far-reaching consequences by hampering our responses towards needy people.

This result supports the psychological numbing hypothesis: repeated exposure to negatively charged messages with a high emotional intensity leads to psychological desensitization. Although this effect has not been demonstrated in relation to hate speech, it has been confirmed for other types of highly emotional stimuli, such as violent media stimuli and violent video-games. In particular, it has been shown that repeated exposure to media violence *decreases* negative affect^51^, playing violent games reduces physiological arousal (heart rate and galvanic skin response) in response to real-life violence^52^.

Although prosocial behavior was not measured in this study, given that previous research has shown that helping involves perspective-taking and is associated with increased activation of the mentalizing network (including the TPJ)^53^, it is plausible that reduced TPJ activation results in disengagement from inferring the cognitive and affective states of others and thus diminishes the propensity to help those in need. It has been demonstrated using an unobtrusive measure of empathy and mentalizing (i.e., neural processes involved in those phenomena), rather than self-report measures—the latter are subject to social desirability pressures, highly common in research on such sensitive issues as intergroup empathy. Further research is needed to test the behavioral consequences of this process.

### Limitations and future directions

Numerous important theoretical questions remain for future research. Further research should resolve whether the observed effect of reduced activity of areas involved in mentalization after exposure to hate speech is long-lasting. For instance, a longitudinal study has found that, despite numerous studies showing a decrease in empathy directly after playing video games, such effects do not persist over time^54^. If a similar relation was valid for negative media content and the decrease in brain activity within the mentalizing hub were transient, the consequences would not be as severe as in a scenario where the effects of such manipulation persist over time. Additionally, it would be interesting to ascertain the effect of political attitudes on patterns of neural activity after exposure to hate speech. As a next step, it would be worthwhile to examine whether, in fact, reduced mentalization decreases prosocial behavior when both in-group and out-group members experience hardship.

There are several limitations to this study. One of the limitations of our study is the sample size. Although it exceeds the median size for experimental fMRI studies in recent years^55^, it is still moderate in light of the between-subject study design used.

Moreover, the sample was not diverse in terms of political attitudes and education. To increase the generalizability of the results and mechanisms investigated, a more diverse sample should be examined. We also cannot rule out that, to some extent, the lack of ingroup bias may be related to low sensitivity in the experimental procedures. Due to time constraints, we chose to use only narratives describing physical pain because numerous previous studies had shown that there is greater empathic arousal in response to physical pain experienced by ingroup individuals compared to outgroup individuals^32^. However, Bruneau's study^45^, for instance, indicates larger effects of intergroup bias on emotional suffering relative to physical pain. Thus, subsequent studies should be expanded to include stimuli that present emotional suffering.

## Conclusion

The omnipresence of hate speech in human environments severely affects people’s attitudes and behavior. Researchers of aggression and intergroup relations have often suggested that frequent exposure to hate speech might lead to prejudice, dehumanization, and lack of empathy toward outgroup members^4,56^. Yet the impairment of empathy among people exposed to hate speech has been proposed only theoretically^4,56–58^, and never directly tested empirically.

In the present experimental study, we aimed to directly tackle this question by analyzing the effects of hate speech exposure on empathy and/or mentalizing. The study suggests that immersion in a hateful environment leads to empathic numbing: people exposed to hate speech have limited ability to attribute the psychological perspective of others, regardless of their group membership.

This initial finding suggests that hate speech affects human functioning beyond intergroup relations. It is well established that after being exposed to hate speech against a specific target group (e.g., gay people), people start dehumanizing them^56^ and their sensitivity to hate speech toward this group is reduced^9^. The present study shows that the effects of hate speech cannot be reduced to a single target group. Exposure to hate speech leads to a more general emotional numbing that affects perspective-taking not only toward certain target minority groups but also toward any other individual, including fellow ingroup members. This very basic psychological transformation is not only important from the perspective of human intergroup relations, but it also shows that hate speech poses a threat to any harmonious human interactions and everyday compassion.

## Supplementary Information

Supplementary Figures.

## Data Availability

Group-level whole-brain results maps are available to download from: https://neurovault.org/collections/WGFWVQDF/. The remaining set of data that support the findings of this study are available on request from the corresponding author.
