# Supplementary material for: Exposure to hate speech deteriorates neurocognitive mechanisms of the ability to understand others’ pain
Source: Sci Rep. 2023 Mar 13;13:4127. doi: 10.1038/s41598-023-31146-1 (PMC10011534; doi:10.1038/s41598-023-31146-1)
Supplement: Supplementary file 1 — Supplementary Figures. [file 41598_2023_31146_MOESM1_ESM.docx]

Supplementary Information

*Insert Figure S1. here*


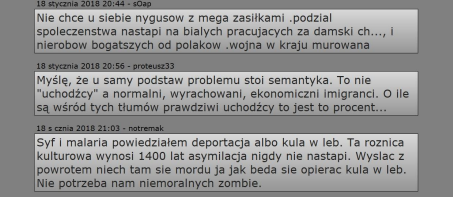
*Figure S1. Sample comments page.*

*Insert Figure S2. here*


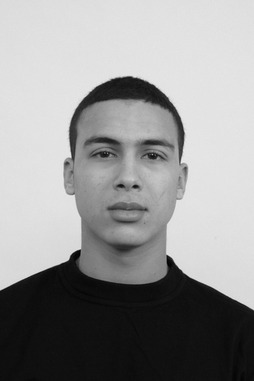

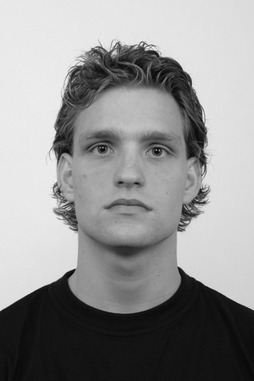


*Figure S2. Examples of images from the Radboud Faces Database* [^[40]^](https://sciwheel.com/work/citation?ids=8565362,892404&pre=&pre=&suf=&suf=&sa=0,0) *used in the study.*
